# Case Report: two cases of anti-neutrophil cytoplasmic antibody-associated vasculitis involving large vessels

**DOI:** 10.3389/fcvm.2024.1434734

**Published:** 2024-07-18

**Authors:** Hanyu Zhang, Dingfang Yan, Yuehua Wei, Yun He, Junjie Chang, Wenjun Zhang

**Affiliations:** ^1^ Department of Medical Ultrasound, Taihe Hospital, Hubei University of Medicine, Shiyan, China; ^2^ Cancer Center, Renmin Hospital of Wuhan University, Wuhan, China

**Keywords:** anti-neutrophil cytoplasmic antibodies, vasculitis, large vessels, echocardiography, computed tomography

## Abstract

Anti-neutrophil cytoplasmic antibody (ANCA)-associated vasculitis (AAV) is a group of systemic diseases caused by a combination of many factors, including genetics, environment, and immunity. AAV is characterized by predominantly small-vessel involvement and has a variety of clinical manifestations. Small-vessel lesions of the kidneys and lungs are common, and lesions of medium-sized arteries may also present, but the involvement of large arteries and their primary branches is very rare. This report delineates two instances of AAV with large arterial involvement, one case presenting with lesions of the aortic valve and the other with lesions of the pulmonary artery. The first case involved a 57-year-old man with no underlying diseases. Transthoracic echocardiography showed thickening of the left and right coronary valves of the aortic valve with enhanced echogenicity, moderate echogenic masses were seen on both valve leaflets, and the leaflets had restricted opening and poor closure. Blood tests showed positive perinuclear anti-neutrophil cytoplasmic antibodies (p-ANCA) and anti-myeloperoxidase (MPO) antibodies. The patient's aortic valve thickening virtually disappeared after treatment with hormones combined with immunosuppressive agents. The second case involved a 60-year-old woman whose transthoracic echocardiography and CT (computed tomography) angiography of the pulmonary arteries showed wall thickening of the main pulmonary artery and the proximal left and right pulmonary arteries, leading to luminal stenosis. Blood tests showed positive cytoplasmic anti-neutrophil cytoplasmic antibodies (c-ANCA) and anti-proteinase 3 (PR 3) antibodies. The patient's pulmonary artery wall thickening reduced after receiving hormones in combination with immunosuppression but she died of heart failure during subsequent treatment. The patient had been diagnosed with tuberculosis six months earlier and had been poorly treated with anti-tuberculosis therapy. The involvement of large arteries in AAV is a rare and critical condition with rapid progression and a high mortality rate. Early recognition of this type of AAV and aggressive immunosuppressive therapy may facilitate the reversal of the vascular lesion and a reduction in the risk of patient death.

## Introduction

AAV is an autoimmune small vessel inflammatory disease associated with the deposition of ANCA, with the primary target antigens being the cytoplasm of neutrophils and monocyte cytoplasmic, which can instigate inflammation and damage across multiple organ systems, notably manifesting in the renal and pulmonary systems (1). AAV is more commonly observed in middle-aged and elderly patients, who often have a variety of underlying disease (2). The initial clinical manifestations of AAV are atypical, frequently presenting with non-specific symptoms such as fever, fatigue, weight loss, and joint and muscle pain, which are easy to be misdiagnosed and missed, the optimal timing of treatment for AAV is often delayed. When lesions occur in multiple systems, the clinical presentation becomes complex and diverse, leading to a poorer prognosis. Cardiac, pulmonary, and renal involvement are high-risk factors for mortality in AAV (3). The 2022 American College of Rheumatology/European Alliance of Associations for Rheumatology classification criteria for AAV state that a single item of ANCA presence accounts for more than 5 points. Combined with other relevant imaging tests and pathology, it is sufficient for diagnosing AAV. This guideline simplifies the diagnostic process of AAV and emphasizes the significance of ANCA testing (4, 5). Systemic vasculitis were classified into seven categories based on the size of the vessels involved, characteristic clinical and histopathologic features, which include large vessel vasculitis, intermediate vessel vasculitis, and small vessel vasculitis (6). Vasculitis of adjacent diameter categories may have overlapped vascular involvement. For example, large vessel vasculitis may present with medium vessel involvement, and medium vessel vasculitis may present with both large and small vessel involvement, but small vessel vasculitis seldom presents with large vessel involvement, especially large artery involvement. AAV is a category of small vessel vasculitis, which mainly involves small vessels in the lungs, kidneys, ear, nose, throat, etc. It can also present with intermediate vessel involvement, but the occurrence of large artery involvement is very rare.

## Case presentation

### Case 1: AAV involving the aortic valve

**Figure 1:**
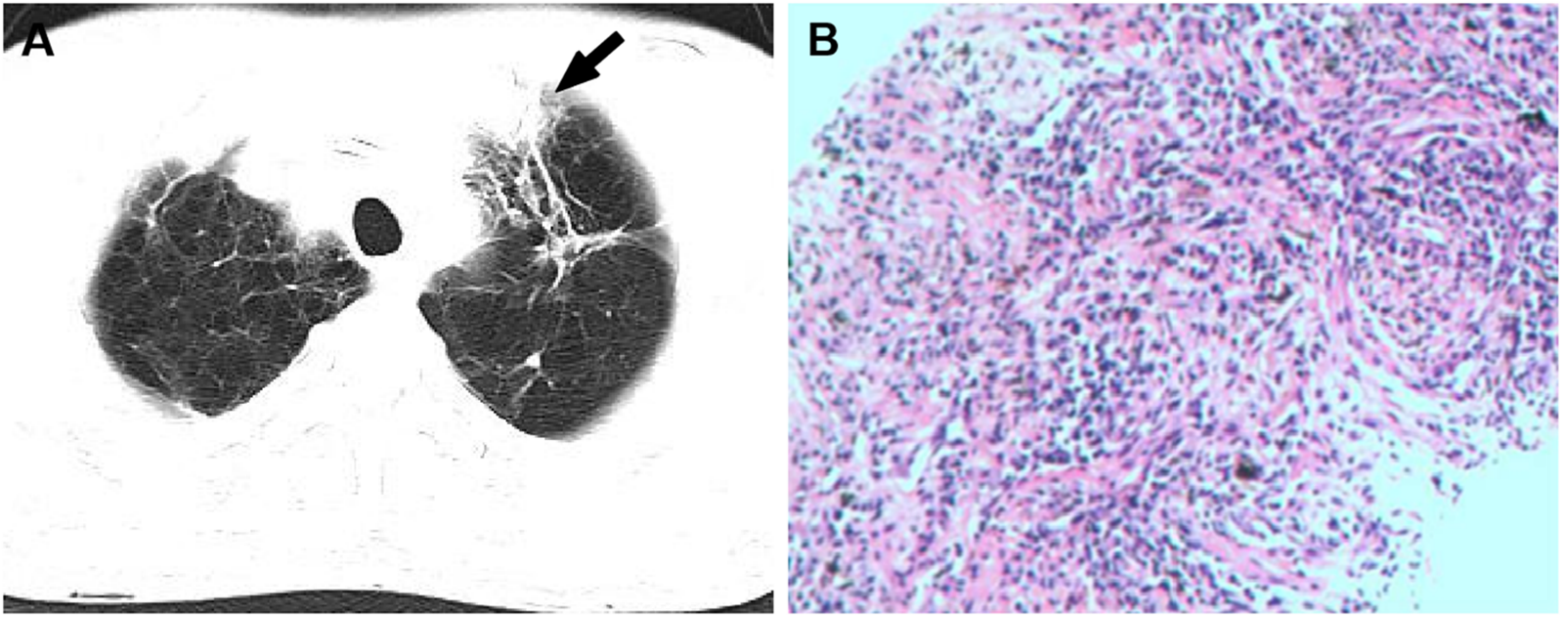
(**A**) the CT image showed a lobulated and spiculated mass (white arrow) in the apical segment of the left upper lobe. (**B**) Hematoxylin and eosin stained paraffin sections showed fibrous connective tissue (at ×200).

**Figure 2:**
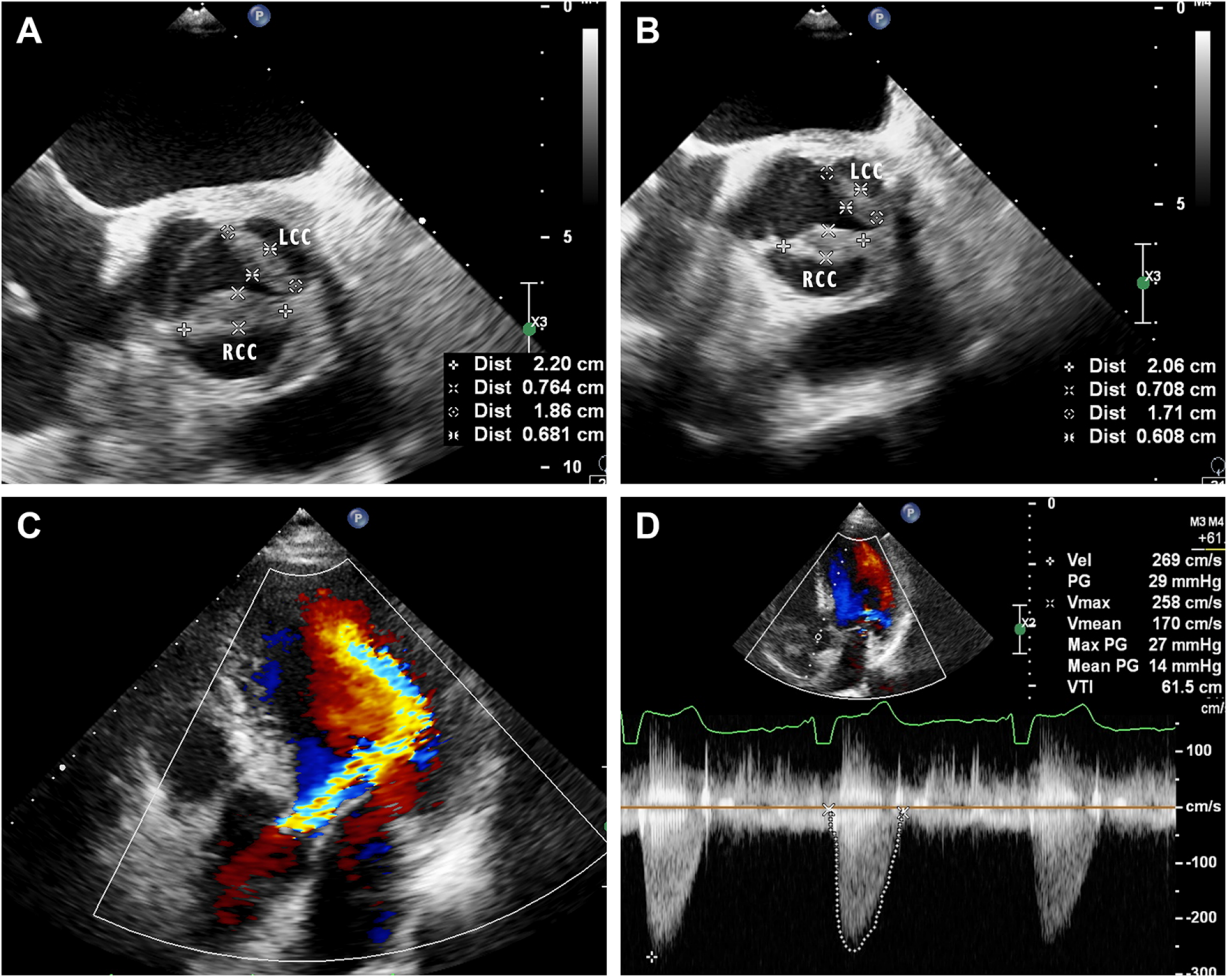
(**A**) Mid-esophageal aortic valve short-axis view of transesophageal echocardiography on admission showed that the left and right coronary valves of the aortic valve were 18.6 × 6.8 mm and 22.0 × 7.6 mm, respectively. (**B**) Mid-esophageal aortic valve short-axis view of transesophageal echocardiography after treatment showed improved thickening of the left and right coronary valves of the aortic valve, which were 17.1 × 6.1 mm and 20.9 × 7.1 mm, respectively. LCC, left coronary cusp; RCC, right coronary cusp. (**C**) Apical five chamber views of transthoracic echocardiography showed moderate regurgitation flow signal during diastole of the aortic valve. (**D**) Color Doppler ultrasound showed accelerated blood flow through the aortic valve during systole.

A 57-year-old male without medical history came to our hospital due to intermittent low-grade fever for 10 days. His body temperature fluctuated between 37.5–38.0°C. On admission, physical examination revealed a temperature of 37.9°C and a heart rate of 94 beats per minute. A diastolic murmur could be heard in the second auscultation area of the aortic valve. Laboratory tests indicated an elevated erythrocyte sedimentation rate (ESR) to 35 mm/h, an increased white blood cell count to 13.56 × 10^9^/L, and a raised percentage of neutrophils to 72.9%. p-ANCA was positive and the anti-MPO antibody exceeded 400 RU/ml (normal value, <20.0 RU/ml). c-ANCA and anti-PR3 antibodies were both negative. Multiple blood cultures showed no bacterial infection, and acid-fast stain test of Mycobacterium tuberculosis was negative. Electrocardiogram showed sinus rhythm. Chest CT revealed a lobulated and spiculated mass in the apical segment of the left lung upper lobe, measuring approximately 4.8cm × 3.7 cm (Figure 1A). Subsequently, the patient underwent CT-guided puncture biopsy of the mass in the left upper lobe. Pathological findings showed predominantly fibrous connective tissue with some alveoli showing organizing pneumonia (Figure 1B). Echocardiography indicated thickening and enhanced echoes of the left and right coronary cusps of the aortic valve, with moderate echogenic masses observed on both valve leaflets. Aortic valve opening was limited and closure was poor (Figure 2A). Color Doppler ultrasound showed accelerated blood flow through the aortic valve during systole, with a peak speed of 2.7 m/s, a pressure gradient of 29 mmHg, and moderate regurgitation flow signal during diastole (Figures 2C,D). The patient was diagnosed with AAV based on laboratory and relevant imaging. Therefore, the patient was treated with prednisone (60 mg/day) and methotrexate (15 mg/week) for 10 days before being discharged. After discharge, the patient continued to take oral steroids combined with immunosuppressants for 2 months. Follow-up chest CT showed the disappearance of the pulmonary mass, and the organizing pneumonia had improved compared to before. Echocardiogram showed improvement in the thickening of the left and right coronary cusps of the aortic valve compared to the previous examination (Figure 2B). p-ANCA and anti-MPO antibodies decreased significantly throughout the treatment process. Comparing the changes in aortic valve lesions before and after treatment, the thickening of the aortic valve of the patient was considered to be inflammatory and hyperplastic changes of the aortic valve due to AAV. After continuing treatment with steroids and immunosuppressants for half a year, the thickening of the aortic valve almost disappeared.

### Case 2: AAV involving the main pulmonary artery and its primary branches

**Figure 3:**
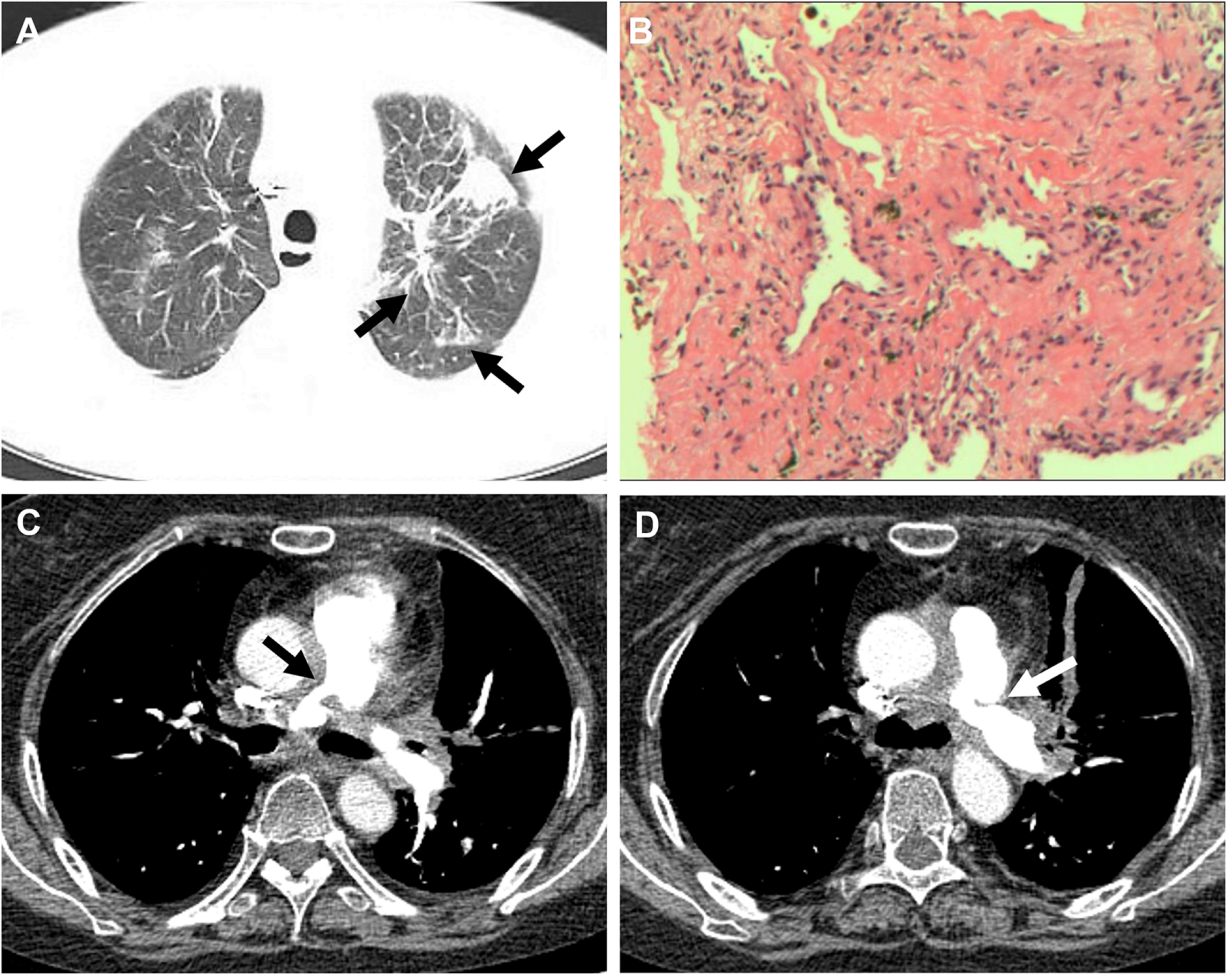
(**A**) CT showed multiple nodular, striated, and mass-like shadows (white arrow) in the left upper lobe of the lung. (**B**) Hematoxylin and eosin stained paraffin sections showed interstitial fibrotic tissue proliferation and scattered few chronic inflammatory cell infiltrates (at ×200). (**C,D**) Pulmonary artery CT angiography showed thickening and narrowing (white arrow) of the proximal walls of the left and right pulmonary arteries.

**Figure 4:**
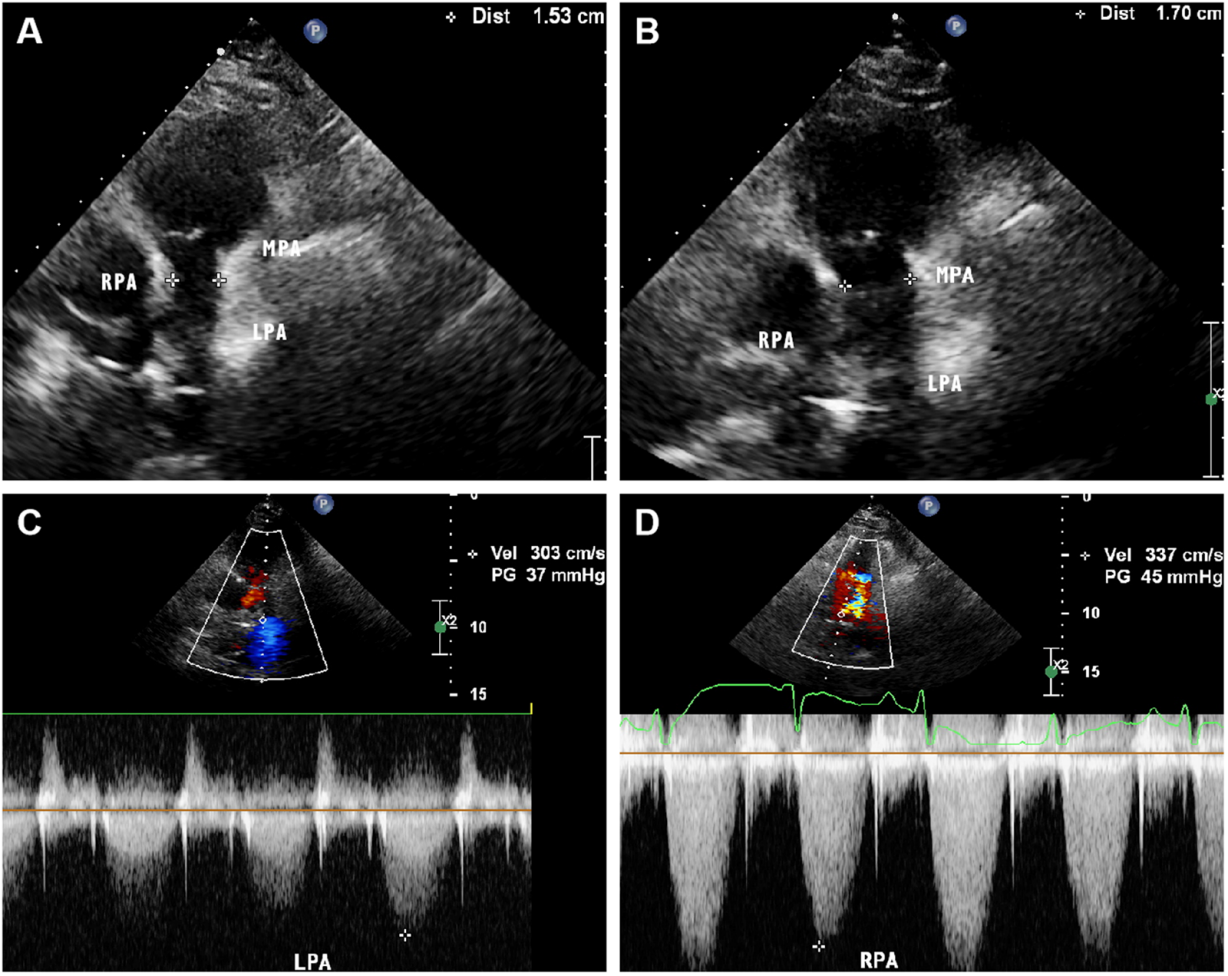
Parasternal pulmonary artery long axis view of transthoracic echocardiography on admission showed narrowing of the main pulmonary artery lumen with an internal diameter of 15 mm. (**B**) Parasternal pulmonary artery long axis view of transthoracic echocardiography after treatment showed a slight improvement in the narrowing of the main pulmonary artery, with an internal diameter of 17 mm. (**C,D**) Color Doppler showed that the systolic blood flow velocity of left and right pulmonary artery was about 3.0 m/s and 3.3 m/s, respectively.

A 60-year-old female presented to our hospital with intermittent fever and cough for 2 months. 6 months before that, the patient came to our hospital with persistent high fever for 1 month. Tuberculosis antibody was positive and three sputum cultures did not reveal antacid bacilli. She underwent fiberoptic bronchoscopy with tissue biopsy. Pathological examination revealed scattered inflammatory cells, macrophages, and no malignant cells were seen. Chest enhancement CT showed multiple nodular, striated, and mass-like shadows in the left lng upper lobe and hilum area of the lung, with mild circumferential enhancement, as well as scattered nodular and striated foci in the rest of the lungs. ^18^F-fluorodeoxyglucose (FDG) positron emission tomography/computed tomography (PET/CT) scan showed abnormally high FDG uptake in the nodules of the upper lobe of both lungs, and also in scattered lymph nodes of the hilar and mediastinal regions. These findings suggested a high probability of pulmonary tuberculosis and then respiratory physicians initiated diagnostic anti-tuberculosis treatment. In addition, the patient was treated with prednisone (20 mg/day) for 15 days due to persistent hyperthermia. Subsequently, this patient was discharged from the hospital with normal body temperature, and she continued to receive 6 months of anti-tuberculosis treatment with standard therapeutic doses of oral isoniazid, rifampicin, ethambutol, and pyrazinamide. After admission, physical examination revealed a temperature of 38.4°C. Systolic murmur could be heard along the left sternal border at the second intercostal space. Auscultation of the lungs revealed coarse breath sounds, with no dry or wet rales heard. Laboratory tests showed an elevated ESR to 37 mm/h and an elevated ultrasensitive C-reactive protein to 4 μg/ml. c-ANCA was positive and the anti-proteinase 3 (PR3) antibody was elevated to 103.5 RU/ml (normal value, <20 RU/ml). Multiple blood and sputum cultures showed no bacterial infection. Antinuclear antibodies, antiphospholipid antibodies, phosphorus cancer-associated antigen, and p-ANCA were all normal. Chest enhancement CT showed no significant changes in lung lesions compared to six months earlier, and stenosis at the beginning of the left and right pulmonary arterial trunks (Figure 3A). Echocardiography showed that the wall of the main pulmonary artery and the left and right pulmonary arteries were unevenly thickened, and the lumen was narrowed (Figure 4A). Color Doppler showed a colorful mosaic pattern of blood flow in the main pulmonary artery and the left and right pulmonary arteries during systole, with systolic blood flow speeds of approximately 3.5 m/s, 3.0 m/s, and 3.3 m/s, respectively (Figures 4C,D). Pulmonary artery CT angiography showed uneven thickness of the main pulmonary artery, thickening and narrowing of the proximal walls of the left and right pulmonary arteries, and occlusion of the branches of the upper left pulmonary artery (Figures 3C,D). All these observations strongly supported the diagnosis of AAV, rather than the pulmonary tuberculosis diagnosed six months before. To validate the diagnosis, the patient underwent a CT-guided puncture biopsy of the left pulmonary nodule. Pathological findings showed interstitial fibrotic tissue proliferation and scattered few chronic inflammatory cell infiltrates, which further supported our diagnosis (Figure 3B). The patient was treated with prednisone (50 mg/day) and cyclophosphamide (75 mg/day) for 15 days. Follow-up echocardiogram showed a reduction in stenosis of the main pulmonary artery and the right and left pulmonary arteries (Figure 4B). This confirmed that hormonal and immunosuppressive treatments can improve pulmonary stenosis. Therefore, we concluded that the pulmonary artery stenosis in this patient was a change caused by AAV. In the subsequent treatment process, the patient's condition worsened, and she died from heart failure despite resuscitation efforts.

## Discussion

AAV encompasses three types of Granulomatosis with Polyangiitis (GPA), Microscopic Polyangiitis (MPA), and Eosinophilic Granulomatosis with Polyangiitis (EGPA) (7). The clinical manifestations of AAV are diverse, which can involve all tissues and organs of the body and blood vessels, and often lead to multi-organ and multi-systems involvement. GPA commonly affects the ear, nose, throat and lungs, with symptoms including purulent bloody nasal discharge, decreased hearing, cough, dyspnea, and hemoptysis, etc. MPA is prominent in renal, lungs, and nervous system involvement, with symptoms such as haematuria, oliguria, oedema, haemoptysis, abdominal pain, sensory abnormalities in limbs, etc. Symptoms of EGPA patients are similar to those in GPA patients, with the distinguishing feature being that nearly all patients also present with asthma or peripheral eosinophilia. Solans et al. reported an overall mortality rate of 28.7% for AAV (8). Late age of onset, multiple organ involvement, especially hemoptysis and cardiovascular system involvement such as cardiac valvulopathy and heart failure, disease activity, severe infections, and previous underlying diseases are all risk factors for death in AAV patients (3). Moreover, relapses are common in AAV, with a relapse rate much higher than that in systemic rheumatic diseases such as SLE. Previous studies have shown that the frequency of recurrence mainly depends on the type of vasculitis, and MPA patients are more prone to recurrence compared to GPA and EGPA (9). Since AAV typically affects multiple organs and has a poor prognosis, with high mortality and relapse rate, clinicians need to integrate clinical symptoms, various imaging studies, and laboratory tests to achieve an early diagnosis and initiate prompt treatment.

Under the influence of various predisposing factors such as genetic background, microbial infections, and drugs, MPO and PR3 are exposed to the immune system, prompting B lymphocytes to produce ANCA. ANCA then binds to the corresponding target antigens and releases a variety of enzymes that cause the rupture of leukocytes, resulting in vascular lesions, which is a pathophysiological factor in the development of AAV and vascular damage (10). Among them, GPA pathogenesis is associated with PR3-ANCA, MPA is associated with MPO-ANCA, and ANCA positivity is low in EGPA, with MPO-ANCA being more common (11). When AAV involves large arteries, ANCA serves as a crucial distinguishing marker between AAV and other conditions such as Takayasu arteritis and giant cell arteritis. Therefore, serological testing for ANCA and regularly monitoring changes in ANCA levels are of significant importance for the diagnosis and treatment of AAV.

We reported two rare cases of AAV involving large vessels, both of which presented with fever. Chest CT scans in both cases revealed lung abnormalities, and etiological examinations were negative, indicating that the conditions were not infectious lesions. The first case involved a male in good health status previously. Ultrasound revealed an aortic valve lesion. Pathological findings of a puncture biopsy of a mass in the upper lobe of the left lung showed predominantly fibrous connective tissue with some alveoli showing mechanised pneumonia changes. Combined with serological ANCA testing (p-pattern by immunofluorescence and MPO antibodies detected by ELISA), the diagnosis was MPA with involvement of the aorta. After treatment, the aortic valve lesion was almost reversed and the lung mass disappeared, and the organizing pneumonia was better than before (Table 1). With promptly diagnosis and quickly targeted therapeutic measures, the patient's condition was effectively controlled with remarkable efficacy.

**Table 1:** Timeline.

Sequence of events	Before the treatment	Treatment for 3 months	Treatment for 6 months
Chest CT	A lobulated and spiculated mass in the apical segment of the left lung upper lobe	Lung masses disappeared	
Echocardiography	The left and right coronary cusps of the aortic valve were thickened, and moderately echogenic masses were seen on both valve leaflets	Improvement in the thickening of the left and right coronary cusps of the aortic valve	The thickening of the left and right coronary cusps of the aortic valve almost disappeared
Medication plan		Oral prednisone (initial dose: 60 mg/day) and methotrexate (15 mg/week) for 3 months	Oral prednisone reduced to 5 mg/day, methotrexate measurement unchanged

**Figure 5:**
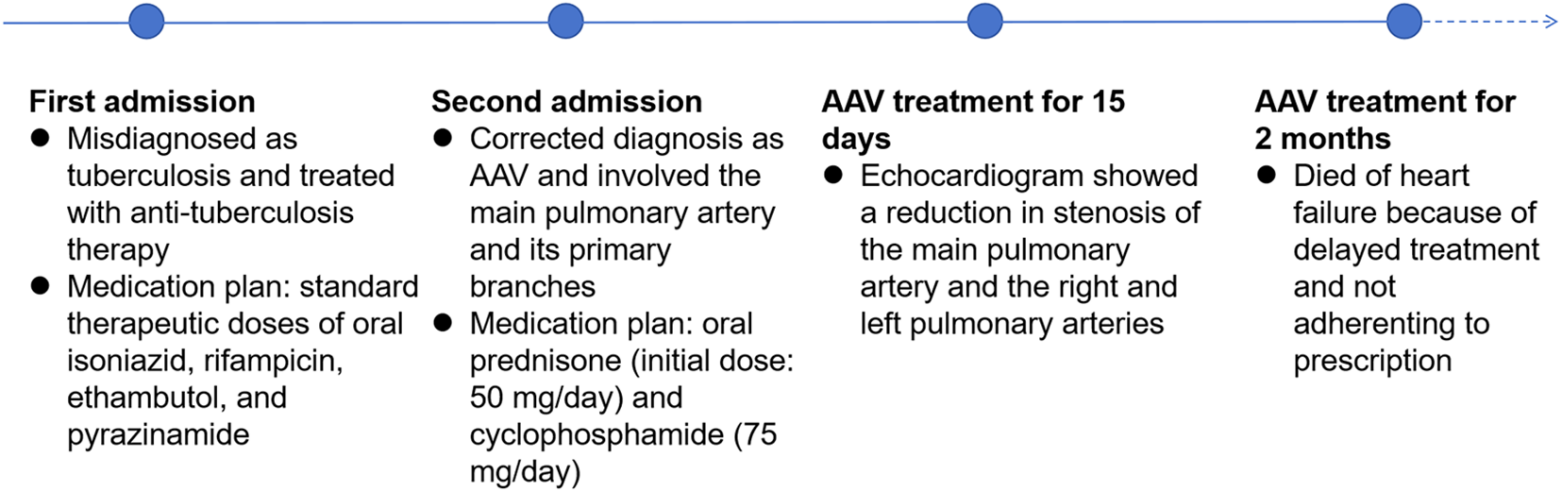
Timeline of the second patient's diagnosis and treatment process.

The second case involved a 60-year-old woman with persistent fever. At the first time of admission six months earlier, although there was no direct pathogenetic evidence of tuberculosis, the chest enhancement CT and PET/CT findings were highly favorable for the diagnosis of tuberculosis. We treated her with diagnostic anti-tuberculosis treatment for a high incidence of tuberculosis in China (12). In addition, short-term, low-dose prednisone therapy, which also has a therapeutic effect on AAV, originally used for her persistent hyperthermia, therefore, her condition was under control and she was free of fever during the hospitalisation. After being discharged, the patient continued to receive anti-tuberculosis treatment for 6 months. At the second time of admission, ultrasonography and pulmonary artery CT angiography both showed wall thickening and luminal narrowing in the main pulmonary artery and proximal left and right pulmonary arteries. Pathological findings of puncture biopsy of the left lung nodule showed interstitial fibrotic tissue hyperplasia and scattered few chronic inflammatory cell infiltrations. Blood tests indicated positive c-ANCA and anti-PR 3 antibodies. Based on these findings, the patient could be diagnosed with GPA, and her condition had worsened compared to six months earlier, as the lesions had involved the main pulmonary artery and its primary branches. After receiving treatment with steroids combined with immunosuppressants, the condition of the pulmonary artery stenosis improved. She died of heart failure eventually because she did not receive timely treatment and was not adherent to prescription. Figure 5 is a timeline of the patient's diagnosis and treatment process. The patient's clinical manifestations lacked specificity, and the imaging findings were more likely to be tuberculosis; in addition, without enough knowledge of AAV, which is clinically rare, non-specialists could not rule out other non-tuberculous lung diseases and autoimmune diseases that start with pulmonary symptoms, resulting of the misdiagnosis and delay of the treatment.

When dealing with the aortitis caused by AAV, it is important to consider the differential diagnosis of other types of aortitis, which include both infectious and non-infectious causes. Non-infectious causes encompass other autoimmune diseases such as sarcoidosis, ankylosing spondylitis, Behçet’ s disease, and Sjögren’ s syndrome, while infectious diseases include endocarditis caused by bacteria, fungi, or syphilis. RY Leavitt et al. introduced the term “polyangiitis overlap syndrome”, which specifically refers to a vasculitis syndrome that features overlapping characteristics of polyarteritis nodosa, EGPA, and primary cutaneous vasculitis (13). However, this term has expanded to include overlaps among distinct vasculitis syndromes. Thus, the presence of large vessel lesions in AAV patients might be interpreted as AAV combined with Takayasu arteritis (TA) or giant cell arteritis (GCA). However, Chirinos et al. reported a retrospective case study of large vessel involvement in AAV, suggesting that large vessel involvement in AAV differs from classic TA and GCA in terms of epidemiology, clinical presentation, and pathological features, thus favoring a diagnosis of AAV. The primary manifestations of large vessel involvement in AAV are aortic aneurysms, periaortitis, and arterial dissection, while TA primarily manifests as diffuse thickening of the vessel wall, which leads to stenosis or occlusion. Moreover, AAV with large vessel involvement rarely presents with symptoms such as jaw claudication, loss of vision, scalp tenderness, and the early rheumatic polymyalgia or temporal arteritis seen in GCA. In addition, the locations of lesions in TA and GCA are primarily in the outer and inner layers of the aorta, whereas the pathological feature of AAV in large vessels is often transmural aortitis (14). Thus, the presence of large vessel involvement in AAV may not be an “overlap” between AAV and Takayasu's arteritis or GCA.

Patients with large vessel involvement in AAV are often in the active phase of the disease and require aggressive treatment. This typically involves high-dose corticosteroids combined with immunosuppressants like cyclophosphamide. Apart from corticosteroids, stronger immunosuppressants such as cyclophosphamide are crucial for inducing remission in AAV (14). If conventional medication does not satisfactorily alleviate the large vessel lesions and significant hemodynamic changes have occurred, surgical intervention may be considered. However, surgery is suitable for patients with stable condition and a maintenance dose of medium to low corticosteroids.

## Conclusion

These two case reports underscore the rarity, complexity and severity of AAV with large vessel involvement. The diagnosis of AAV involving large vessels requires a combination of multiple imaging methods and serological examination, which poses a great challenge. Understanding such rare presentations can aid clinicians in recognizing and diagnosing similar atypical cases more effectively. Although its prognosis is poor, vascular lesions can be reversed if treated early. Therefore, clinicians should diagnose AAV as early as possible, thoroughly evaluate the functional status of the affected organs, provide rational treatment, follow up closely, and adjust the treatment plan timely.

## Data Availability

The original contributions presented in the study are included in the article/Supplementary Material, further inquiries can be directed to the corresponding author.
